# Psychometric properties of a French version of a Dutch scale for assessing breast and body image (BBIS) in healthy women

**DOI:** 10.1186/1472-6874-13-24

**Published:** 2013-05-16

**Authors:** Noémie Resseguier, Catherine Noguès, Roch Giorgi, Claire Julian-Reynier

**Affiliations:** 1 INSERM, U912, SESSTIM, Marseille, France; 2 IRD, U912, SESSTIM, Marseille, France; 3 Aix-Marseille Université, U912, SESSTIM, Marseille, France; 4 Hôpital René Huguenin, Saint-Cloud, France; 5 Institut Curie, Paris, France; 6 Institut Paoli-Calmettes, Marseille, France

**Keywords:** Body image, Women, Breasts, *BRCA1/2* mutation, Psychometric properties

## Abstract

**Background:**

Genetic testing among women for *BRCA1/2* mutation can have various psychological effects, such as those focusing on body image. The aim of this study was to examine the psychometric properties of a generic scale assessing breast and body image (BBIS) in healthy women tested for *BRCA1/2* mutations.

**Methods:**

A Dutch body image scale focusing on both general and breast-related body image was translated into French. It was presented to a French cohort of female cancer-free *BRCA1/2* mutation carriers and non-carriers (N = 568). The psychometric properties of the scale were studied by assessing its dimensional and factorial structure, internal consistency, construct-related validity, and external validity.

**Results:**

The scale was found to be a satisfactory psychometric tool for assessing both body image and breast image. The three main dimensions which emerged were classified under the headings “values attached to body image”, “satisfaction with body image and perceived attractiveness”, and “satisfaction with breasts”. The BBIS scores were not significantly associated with the participants’ socio-demographic characteristics or their *BRCA1/2* mutation carrier status, but significant associations were observed between these scores and the women’s medical and behavioural characteristics.

**Conclusions:**

The BBIS is a generic tool which can be used to assess body image in either affected or unaffected women. The scale will have to be administered to other populations in order to confirm its validity.

## Background

Body image is a complex, multidimensional concept at the crossroads between various fields. It involves people’s self-perceptions and their attitudes (*i.e.*, their thoughts, feelings, and behavior) towards their body, and suitable tools are required for assessing it [1-3]. As suggested in a previous cognitive-behavioral model of body image, it includes (i) body image evaluation which refers to satisfaction or dissatisfaction with one’s body, including evaluative beliefs about it, and (ii) body image investment which refers to the cognitive, behavioral, and emotional importance persons attach to their appearance [2]. Body image assessment has been described as stemming from the degree of discrepancy or congruence between self-perceived physical characteristics and personally valued ideals of physical appearance [4]. Research on body image focused initially on female populations. Since body image is experienced differently among men and women [5,6], scales assessing body image should be gender-related. In women, body image relates to femininity, and the latter aspect should also be assessed when measuring women’s body image [7,8]; since the breasts are one of the main symbols of femininity, body image scales should include how they are perceived, whatever the context involved (clinical populations with and without breast disease and general populations).

In clinical practice, body image is a useful concept for assessing the effectiveness of surgical and medical interventions (*e.g.,* plastic surgery [9,10], dieting for obesity [11-13], and treatment of eating disorders [14,15]). Physical diseases and injuries and psychiatric disorders and their treatment can completely change the functional integrity of the body and its appearance, which in turn can greatly affect patients’ body image, their psychosocial wellbeing and their quality of life. In order to assess body image in clinical populations, several scales have been developed for use in clinical practice and research and validated on these particular populations [16-18]. However, these scales can be used only on the specific populations for which they were designed, to assess the effects of a disease and its treatment on the patients’ body image.

The factors involved in the case of healthy subjects are likely to differ completely from those contributing to the body image of affected patients [3,19,20]. Questions about the effects of disease and its treatment on respondents’ body image are no longer relevant here, and many of the previously developed scales are unsuitable for use in this context. Greater attention should be paid to developing generic body image scales which could be used on healthy populations. Generic scales would be particularly useful in the context of longitudinal studies, as they could be used to repeat the measurements over a period of time, even if the subjects’ condition has evolved. Items relating to highly specific situations could be added to these generic scales when necessary in order to obtain both a generic body image and a specific body image, depending on the framework of the survey.

Halfway between clinical and healthy populations, there exists a group consisting of people at risk, who may possibly contract a disease one day or may have to make prophylactic decisions [21-23]. A typical example of populations of this kind is that consisting of carriers of deleterious genetic mutations, such as *BRCA1/2* mutations. Women with a *BRCA1/2* mutation have up to an 87% lifetime risk of developing breast cancer and a 15-60% lifetime risk of developing ovarian cancer [24,25]. Being a carrier of a *BRCA1/2* mutation has psychosocial effects, including those focusing on how people view themselves such as body image [26]. In addition, some of the women with a high genetic risk of developing cancer undergo prophylactic surgery (risk-reducing mastectomy or oophorectomy), which is liable to have negative effects on these patients’ body image [22,23].

A scale was previously developed by Lodder *et al.* for assessing body image in unaffected women carriers of a *BRCA1/2* mutation [22]. In this study, Lodder established that mutation carriers who underwent prophylactic mastectomy (with reconstruction) had a poorer breast-related image after one year of follow-up than mutation carriers who had opted for surveillance and non-mutation carriers. But as far as we know, this scale has been used but never validated so far [27]. The aim of the present study was therefore to present this generic body image scale and to study its psychometric properties on unaffected French female *BRCA1/2* mutation carriers and non-carriers.

## Methods

### Ethics statement

The informed consent of each participant was obtained at the beginning of the study after explaining the purpose of this study in detail. The study was approved by the French National Commission for Data Protection and Privacy (“Commission Nationale de l’Informatique et des Libertés”).

### Study population

In the framework of the ongoing French GENEPSO (“Gene Etude Prospective Sein Ovaire”) project managed by the French Cancer Genetic Network, *BRCA1/2* mutation carriers were recruited in a routine consultation context at cancer genetic clinics between 2000 and 2006. Non carriers from families where a *BRCA1/2* mutation had been identified were included as well as carriers.

Eligible subjects were therefore women aged 18 years or more, who were cancer-free, belonged to a family in which a deleterious predisposing *BRCA1/2* mutation had been identified, and were tested for this mutation.

### Procedure

Women included in the cohort filled in a self-administered questionnaire at the cancer genetic clinic before delivery of the genetic test results (questionnaire Q_D0_) and a different self-administered questionnaire which was sent to their homes 15 days after delivery of the results (questionnaire Q_D15_). If no answer had been received one month after mailing the questionnaire, a reminder and a copy of the questionnaire were sent out. All the completed questionnaires were mailed back to the coordinating centre. The cancer geneticists also completed a questionnaire describing the women’s family members and their medical characteristics at inclusion and follow-up.

### Instruments

The questionnaire Q_D0_ focused on the respondents’ sociodemographic data (on aspects such as age, marital status and education). The questionnaire Q_D15_, which focused on their psychological characteristics [27], assessed the respondents’ depressive symptoms using the French version of the Center for Epidemiologic Studies Depression Scale (CES-D) [28,29] and their breast and body image. The CES-D scale consists of 20 items giving a total score after giving each item a score of 0/1/2/3. The overall score was dichotomized using the value of 23 as a cutoff point, as previously recommended for identifying French women with high depressive symptoms [28].

At the onset of the study, no questionnaires on body image tailored to the present study population were available and validated in French. We translated the body image scale presented by Lodder *et al.* in a similar study [22]; it was first translated by two French native speakers who were fluent in Dutch, before being translated back into Dutch by a Dutch native speaker who was fluent in French [30]. To develop this scale, Lodder followed previous recommendations (Hopwood [16,17]) by addressing both the question of general body image and that of breast-related body image. This scale included various aspects which have been said to be important factors contributing to cancer patients’ body image [16]: 1) satisfaction with appearance when dressed, 2) feeling feminine, 3) satisfaction with appearance when naked, 4) feeling attractive, and 5) feeling conscious about one’s appearance. Three questions were added to obtain a specific breast-related body image scale assessing whether women were satisfied with the way their breasts felt when touching them (two items) and with their appearance (one item). Lastly, two other items were included about the importance attached to physical appearance. We ourselves added an item about the importance attached to the appearance of the breasts (item 2). The complete scale therefore consisted of 11 items translated from Lodder’s scale and one item added by ourselves. Among the twelve items on the scale, 10 were positively-worded and two were negatively-worded (reversed coding). A five-point Likert scale ranging from zero to four was used to define the responses in terms of agreement (“Strongly disagree”, “Disagree”, “Neither agree nor disagree”, “Agree”, “Strongly agree”). The list of items is presented in Table 1. We named this scale BBIS, which stands for Breast and Body Image Scale.

**Table 1:** List of the items on the breast and body image scale

**French version**		
Item 1	D’une manière générale	Pour moi, l’apparence physique est importante
Item 2		Pour moi, l’apparence des seins est importante
Item 3		Je soigne beaucoup mon apparence
Item 4	Au cours du dernier mois	J’étais satisfaite de mon apparence quand j’étais habillée
Item 5		Je me sentais très féminine
Item 6		J’étais très consciente de mon image
Item 7		J’étais satisfaite de mon apparence quand j’étais nue
Item 8		J’avais du mal à me regarder nue (inversé)
Item 9		J’avais du mal à toucher ma poitrine (inversé)
Item 10		J’étais satisfaite de l’apparence de mes seins
Item 11		Mes seins étaient agréables au toucher
Item 12		Je me sentais séduisante
English version [22]		
Item 1	Generally speaking	I find it important to look good
Item 2		I find it important that my breasts look good
Item 3		I pay much attention to my appearance
Item 4	In the past month	I was satisfied with my appearance when dressed
Item 5		I felt quite feminine
Item 6		I felt very conscious about my appearance
Item 7		I was satisfied with my appearance when undressed
Item 8		I had difficulty in looking at my body when undressed (reverse coding)
Item 9		I had difficulty in touching my breasts (reverse coding)
Item 10		I was satisfied with the appearance of my breasts
Item 11		My breasts felt pleasant
Item 12		I felt attractive

### Statistical analysis

The characteristics of the study population were described in the population as a whole and in two sub-populations, defined in terms of whether or not there were missing values in the body image scale. The distribution of these characteristics was compared between these two sub-populations using chi-square tests. Determinants for being an incomplete responder to the BBIS were tested using a multivariate logistic regression model. No variable selection was performed using statistical criteria, and adjusted odds ratios were estimated.

Analyses of the frequency distribution were performed at the item level, including missing values. The following analyses were then performed on complete cases.

Pearson’s correlation coefficients between the various items were calculated in order to detect any redundant items.

The dimensionality of the scale was determined by performing exploratory factor analysis (Principal Component Analysis; PCA). The Scree plot and the Kaiser criterion were used to decide about the appropriateness of the number of factors retrieved. A varimax rotation was then performed in order to estimate the factor loadings, and each item was taken to contribute to the factor corresponding to its highest loading value. As the Kaiser criterion tends to result in the over-extraction of factors, we compared the goodness of fit of the selected model with models including fewer factors. We studied five fit indices [31]: the Normed Fit Index (NFI), the Tucker-Lewis Index (TLI), the Comparative Fit Index (CFI), the Root Mean Square Error of Approximation and its confidence interval (RMSEA), and the Bayesian Information Criterion (BIC).

Each factor which emerged was used to define a sub-scale. The score obtained on each sub-scale was calculated by summing the responses to the various items included in the corresponding sub-scale. As an exploratory endpoint, an overall score was also calculated by summing together the scores obtained on the various sub-scales.

The internal consistency was assessed by determining Cronbach’s alpha coefficients. Confidence intervals were determined using bootstrapping methods. Pearson’s correlation coefficients between the various sub-scales were calculated and compared with Cronbach’s α coefficients. If the value of the correlation coefficient was lower than that of the Cronbach’s α coefficients, the components were taken to measure other aspects [32]. The construct-related validity was determined by assessing the item convergent validity and the item discriminant validity.

In order to assess the external validity of the scale, we studied the associations existing between the scores obtained on the BBIS and various covariates previously found in the literature to be associated with the body image. Means BBIS scores were compared in terms of these characteristics, using ANOVA tests.

All tests were two-sided, and differences were taken to be significant at *p-values* < .05. All analyses were performed using the R software.

## Results

### Study population

Among the 613 women who were recruited, 45 did not complete the Q_D15_ questionnaire, including one who declared that she did not want to participate in the study any longer. Data were analysed on the 568 women who answered the Q_D15_ questionnaire (245 *BRCA1/2* mutation carriers and 323 *BRCA1/2* non carriers). Socio-demographic and medical characteristics of the overall study sample and those of the subsamples of women who had not (N = 517, 91%) and those who had (N = 51, 9%) missing values in the BBIS are given in Table 2. Univariate analysis showed that age, level of education and BMI index classes were significantly associated with the presence of missing values in the BBIS. After systematically adjusting on all the covariates collected in a multivariate logistic regression model, only a high level of education was found to be significantly associated with a lower risk of missing values in the BBIS.

**Table 2:** Socio-demographic and medical characteristics of the overall study sample (N = 568) and those of the subsamples of women whose responses to the Breast and Body Image Scale (BBIS) were complete (N = 517) and those whose questionnaires had missing values (N = 51)

	**All women N (%)**	**Women with no MVs in the BBIS N (%)**	**Women with MVs in the BBIS N (%)**	**Univariate analysis**	**Multivariate analysis**		
				**p-value**^**a**^	**adj OR**^**b**^	**CI 95%**	**p-value**
Age (yrs)				<0.01			0.44
≤30	105 (18.5)	102 (19.7)	3 (5.9)		0.29	0.04 – 1.27	
31-40	205 (36.1)	195 (37.7)	10 (19.6)		0.62	0.22 – 1.71	
41-50	152 (26.8)	136 (26.3)	16 (31.4)		0.75	0.30 – 1.93	
>50	106 (18.7)	84 (16.2)	22 (43.1)		1	-	
Level of education				<0.01			<0.01
Less than high school certificate level	139 (24.5)	111 (21.5)	28 (54.9)		1	-	
High school certificate	110 (19.4)	103 (19.9)	7 (13.7)		0.29	0.09 – 0.80	
Above high school level certificate	316 (55.6)	301 (58.2)	15 (29.4)		0.24	0.09 – 0.60	
*MVs*	*3 (0.5)*	*2 (0.4)*	*1 (2.0)*				
Living with a partner				0.22			0.28
No	127 (22.4)	119 (23.0)	8 (15.7)		1	-	
Yes	426 (75.0)	384 (74.3)	42 (82.4)		1.79	0.65 – 6.25	
*MVs*	*15 (2.6)*	*14 (2.7)*	*1 (2.0)*				
Body Mass Index class				0.02			0.70
<18.5 Underweight	33 (5.8)	30 (5.8)	3 (5.9)		1.83	0.39 – 6.26	
18.5-24.9 Normal weight	379 (66.7)	354 (68.5)	25 (49.0)		1	-	
25.0-29.9 Overweight	116 (20.4)	97 (18.8)	19 (37.3)		1.04^*^	0.44 –^*^2.34	
≥30 Obesity	26 (4.6)	24 (4.6)	2 (3.9)				
*MVs*	*14 (2.5)*	*12 (2.3)*	*2(3.9)*				
Regular gynecological follow-up				0.18			0.61
No	58 (10.2)	50 (9.7)	8 (15.7)		1	-	
Yes	506 (89.1)	463 (89.6)	43 (84.3)		0.72	0.16 – 2.33	
*MVs*	*4 (0.7)*	*4 (0.8)*	*0 (0.0)*				
Breast self-examination				0.75			0.44
No	303 (53.3)	277 (53.6)	26 (51.0)		1	-	
Yes	257 (45.2)	233 (45.1)	24 (47.1)		0.75	0.35 – 1.57	
*MVs*	*8 (1.4)*	*7 (1.4)*	*1 (2.0)*				
Depressive symptoms (score CES-D ≥ 23) (Q_D15_)				0.57			0.38
No	434 (76.4)	405 (78.3)	29 (56.9)		1	-	
Yes	96 (16.9)	88 (17.0)	8 (15.7)		1.49	0.59 – 3.46	
*MVs*	*38 (6.7)*	*24 (4.6)*	*14 (27.5)*				
*BRCA1/2* mutation				0.77			0.26
Non carriers	323 (56.9)	293 (56.7)	30 (58.8)		1	-	
Carriers	245 (43.1)	224 (43.3)	21 (41.2)		1.52	0.73 – 3.19	

MVs: missing values; CES-D: Center for Epidemiologic Depression Scale; Q_D15_: questionnaire filled in 15 days after test result disclosure.

^*^The body mass index classes “25.5-29.9 Overweight” and “≥30 Obesity” were pooled to obtain more accurate estimates.

^a^ p-value in univariate tests comparing the distributions of the sociodemographic and medical characteristics according to the status MVs / no MVs in the BBIS.

^b^ Adjusted odds ratio of the excess risk of having missing values in the BBIS.

### Distribution of the responses to the various items on the scale

The distributions of the responses of all the participants (N = 568) to the various items in the BBIS are presented in Table 3. The rate of missing values among the items was low (range: 1.6% - 3.9%). No floor effect was observed. A ceiling effect was observed in the case of the eighth item (reverse coding) and especially in that of the ninth item (reverse coding). The negative wording of these two items may account for the presence of these different distributions. These distributions were actually not very surprising, since there were only a few possible responses to each question.

**Table 3:** Matrix of correlations between the various items on the breast and body image scale 15 days after test result disclosure (N = 517), and distribution of the responses to the various items on the questionnaire (N = 568; bottom of the Table)

	**1**	**2**	**3**	**4**	**5**	**6**	**7**	**8**	**9**	**10**	**11**	**12**
1: It’s important to look good	1											
2: It’s important that breasts look good	0.59	1										
3: Attention to appearance	0.55	0.40	1									
4: Satisfied with appearance when dressed	0.30	0.19	0.42	1								
5: Feeling feminine	0.28	0.23	0.45	0.73	1							
6: Conscious about one’s appearance	0.23	0.22	0.36	0.38	0.49	1						
7: Satisfied with one’s appearance when undressed	0.19	0.11	0.32	0.58	0.55	0.34	1					
8: Difficulty in looking at one’s body when undressed (R)	0.02	−0.01	0.12	0.33	0.28	0.14	0.42	1				
9: Difficulty in touching breasts (R)	−0.03	−0.01	−0.03	0.09	0.12	0.12	0.10	0.29	1			
10: Satisfied with appearance of breasts	0.16	0.19	0.20	0.36	0.39	0.25	0.43	0.21	0.17	1		
11: Breasts feel pleasant	0.19	0.23	0.23	0.32	0.37	0.27	0.32	0.22	0.29	0.53	1	
12: Feeling attractive	0.27	0.23	0.35	0.60	0.63	0.37	0.60	0.41	0.21	0.52	0.54	1
Mean score	3.28	3.22	2.95	2.89	2.92	2.99	2.18	3.00	3.41	2.73	2.92	2.56
Standard Deviation	0.83	0.90	0.90	1.00	1.01	0.96	1.21	1.21	1.13	1.15	1.02	1.03
Median	3	3	3	3	3	3	2	3	4	3	3	3
“Strongly disagree”: N (%)	4 (1)	6 (1)	6 (1)	11 (2)	8 (1)	10 (2)	50 (9)	26 (5)	27 (5)	30 (5)	10 (2)	13 (2)
“Disagree”: N (%)	25 (4)	30 (5)	42 (7)	54 (10)	58 (10)	34 (6)	132 (23)	55 (10)	25 (4)	58 (10)	35 (6)	75 (13)
“Neither agree nor disagree”: N (%)	38 (7)	53 (9)	81 (14)	82 (14)	83 (15)	90 (16)	119 (21)	84 (15)	50 (9)	106 (19)	144 (25)	158 (28)
“Agree”: N (%)	234 (41)	216 (38)	276 (49)	243 (43)	229 (40)	228 (40)	173 (30)	115 (20)	44 (8)	192 (34)	159 (28)	194 (34)
“Strongly agree”: N (%)	256 (45)	251 (44)	154 (27)	164 (29)	176 (31)	184 (32)	79 (14)	270 (47)	404 (71)	162 (29)	198 (35)	106 (19)
Missing value: N (%)	11 (2)	12 (2)	9 (2)	14 (2)	14 (2)	22 (4)	15 (3)	18 (3)	18 (3)	20 (4)	22 (4)	22 (4)

R: Reverse coding.

### Correlations between items on the scale

The Pearson’s correlation coefficients between items are presented in Table 3 (based on fully completed questionnaires, N = 517). The maximum value of these coefficients was 0.73. A few negative correlations were obtained, but since the coefficients were small in these cases, all the items on the scale could be taken to measure a common concept.

### Dimensional structure of the scale

**Figure 1:**
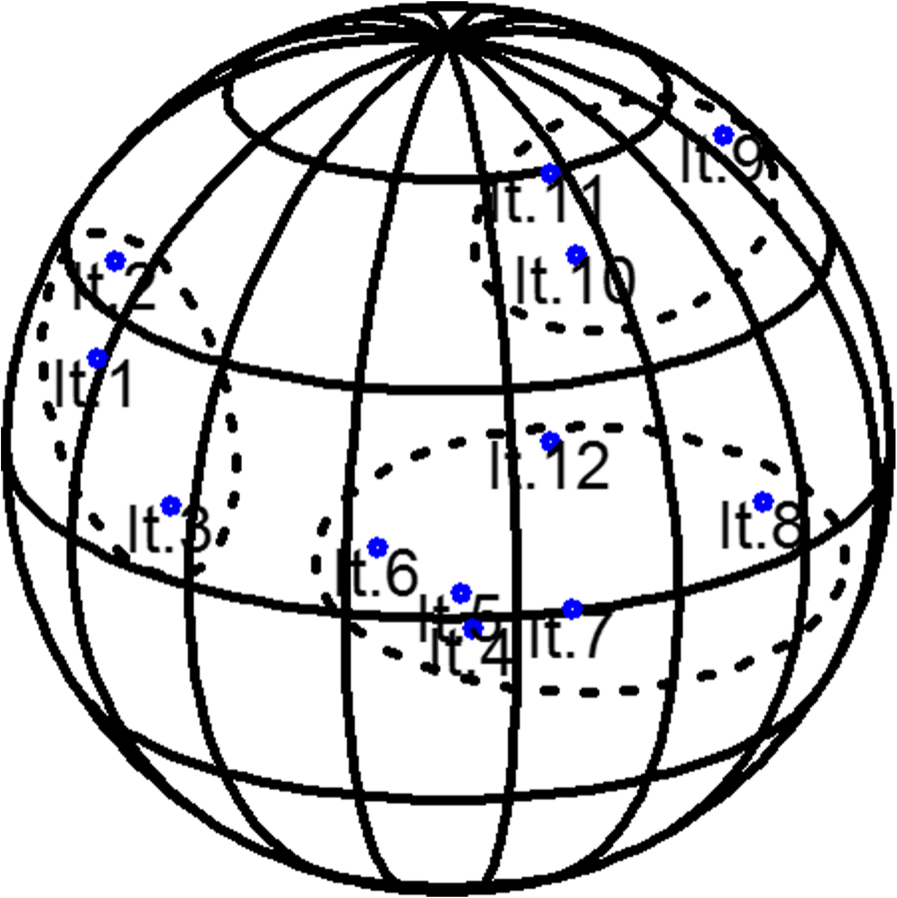
**Three-dimensional diagram of the correlation matrix among the various items on the scale (N = 517).** All items on the scale could be depicted on the same side of the sphere.

**Figure 2:**
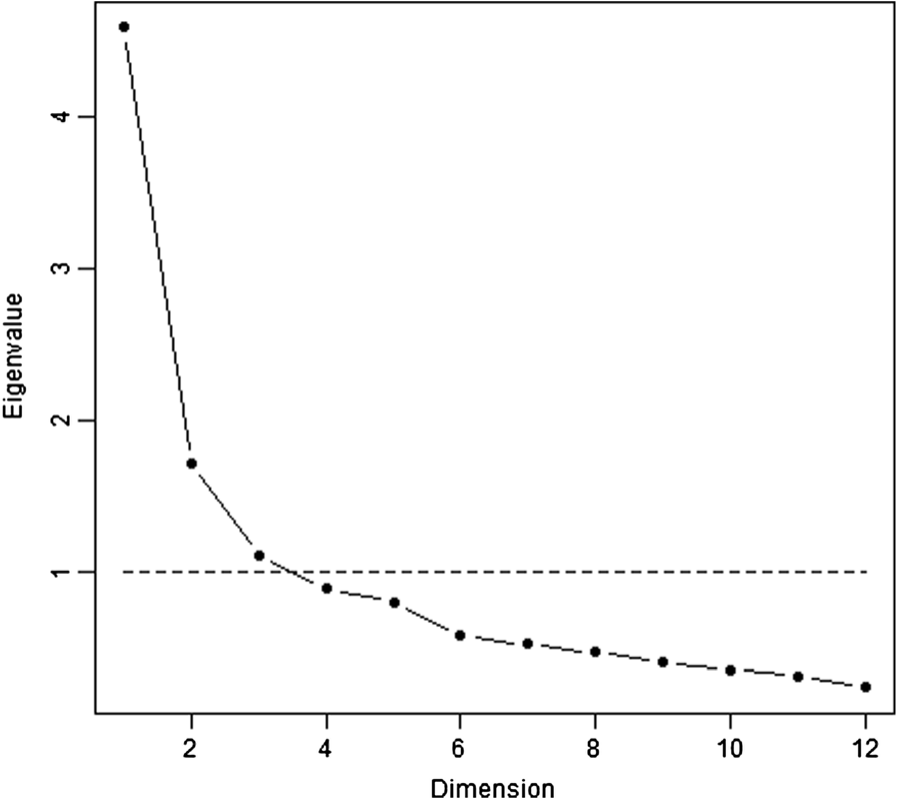
Scree plot of the eigenvalues obtained in the principal component analysis (N = 517).

The three-dimensional representation of the correlation matrix (between items) based on the Principal Component Analysis is presented in Figure 1. Two and three components accounted for 53% and 62% of the variance, respectively. In view of the three-dimensional spherical form of presentation, there seem to exist three dimensions on the scale. The scree plot of the eigenvalues indicated that there may be either one or three dimensions on the scale (Figure 2). Based on Kaiser’s criterion, a three-factor model was used for the factor analysis.

Loading values based on the factor analysis performed with a three-factor model are presented in Table 4. Items 1, 2 and 3 were taken to form the first factor, items 4, 5, 6, 7, 8 and 12, the second factor, and items 9, 10 and 11, the third factor. Based on this loading pattern, factor 1 was labelled “values attached to body image” (“ValBI”), factor 2, “satisfaction with body image and perceived attractiveness” (“SatBIPA”) and factor 3, “satisfaction with breasts” (“SatBr”).

**Table 4:** Loading values obtained in the factorial analysis after varimax rotation with a three factor model (N = 517) (loading values under .10 were not reported)

	**Factor1**	**Factor2**	**Factor3**
Item 1: It’s important to look good	**0.822**	0.153	
Item 2: It’s important that breasts look good	**0.701**		0.107
Item 3: Attention to one’s appearance	**0.579**	0.387	
Item 4: Satisfied with one’s appearance when dressed	0.203	**0.807**	0.145
Item 5: Feeling feminine	0.223	**0.792**	0.205
Item 6: Conscious about one’s appearance	0.237	**0.433**	0.159
Item 7: Satisfied with one’s appearance when undressed		**0.644**	0.303
Item 8: Difficulty in looking at one’s body when undressed (R)		**0.373**	0.288
Item 9: Difficulty in touching one’s breasts (R)			**0.368**
Item 10: Satisfied with appearance of breasts	0.150	0.297	**0.593**
Item 11: Breasts feel pleasant	0.210	0.207	**0.702**
Item 12: Feeling attractive	0.203	**0.604**	0.532

R: Reverse coding.

Three decreasingly complex three- to one-factor models were fitted, and the three-factor model showed the best fit according to the various goodness-of-fit indices: the NFI was 0.95 (0.87 and 0.72 for the two- and one-factor models, respectively), the TLI was 0.92 (0.82 and 0.68), the CFI was 0.96 (0.88 and 0.74), the RMSEA was 0.073 [90% CI: 0.059 - 0.086] (0.110 [90% CI: 0.098 - 0.121] and 0.147 [90% CI: 0.136 - 0.156]), and the BIC was −84.73 (40.34 and 311.96).

### Reliability of the scale

Cronbach’s alpha coefficient on the overall scale was 0.84 (95% CI: [0.81 - 0.85]. The values obtained on the “ValBI”, “SatBIPA” and “SatBr” sub-scales were 0.76 [0.70 - 0.80], 0.83 [0.81 - 0.85] and 0.59 [0.52 - 0.65], respectively.

The value of each inter-sub-scale correlation coefficient was lower than the corresponding Cronbach’s alpha coefficients. All the items on each of the three sub-scales met both the convergent validity and discriminant validity criteria.

### Scores and sub-scores, and associations with participants’ characteristics

Descriptive statistics on the overall score and the various sub-scores are presented in Table 5. The mean overall score was 34.9 (sd: 7.4), and the scores on the dimensions “ValBI”, “SatBIPA”, “SatBr” were 9.4 (sd: 2.2), 16.5 (sd: 4.7) and 9.0 (sd: 2.5), respectively. The various sub-scores are presented in terms of the participants’ socio-demographic and medical characteristics in Table 6. No significant associations were found to exist between the sub-scores on the BBIS and age, level of education, or living with a partner. A higher body mass index was significantly associated with a lower score on the dimensions “ValBI” and “SatBIPA”, but not with the dimension “SatBr”. Having a regular gynecological follow-up and performing breast self-examination were significantly associated with higher scores on each of these dimensions. Depressive symptoms were significantly associated with higher scores on “ValBI” but with lower scores on “SatBIPA” and “SatBr”. No significant associations were found to exist between the scores obtained on the BBIS and carrying a *BRCA1/2* mutation.

**Table 5:** Descriptive statistics on the overall score and the various sub-scores (N = 517)

	**Mean**	**SD**	**Minimum**	**1st Quartile**	**Median**	**3rd Quartile**	**Maximum**
Overall score	34.9	7.4	12	30	36	40	48
Values attached to body image	9.4	2.2	0	8	10	11	12
Satisfaction with body image and perceived attractiveness	16.5	4.7	2	13	17	20	24
Satisfaction with breasts	9.0	2.5	1	7	10	11	12

**Table 6:** Overall score and the various sub-scores in terms of respondents’ socio-demographic and medical characteristics (N = 517)

		**Values attached to body image**			**Satisfaction with body image and perceived attractiveness**			**Satisfaction with breasts**		
	**N**	**Mean**	**Sd**	**p-value**	**Mean**	**Sd**	**p-value**	**Mean**	**Sd**	**p-value**
Age (yrs)				0.67			0.59			0.23
≤ 30	102	9.34	1.78		16.52	4.89		9.25	2.32	
31-40	195	9.23	2.14		16.21	4.73		8.89	2.53	
41-50	136	9.52	2.37		16.65	4.80		8.94	2.55	
>50	84	9.64	2.35		16.93	4.28		9.17	2.26	
Level of education				0.64			0.44			0.88
Less than high school certificate level	111	9.58	2.66		17.23	5.10		9.10	2.73	
High school certificate	103	9.44	2.36		16.74	4.75		9.05	2.36	
Above high school certificate level	301	9.31	1.89		16.15	4.51		8.98	2.39	
Living with a partner				0.88			0.80			0.92
No	119	9.38	2.29		16.37	4.75		9.03	2.36	
Yes	384	9.41	2.14		16.49	4.76		9.01	2.50	
Body mass index class				<0.01			<0.01			0.92
<18.5 Underweight	30	9.70	2.02		19.03	4.24		8.97	2.37	
18.5-24.9 Normal weight	354	9.49	2.07		16.69	4.51		9.05	2.43	
25.0-29.9 Overweight	97	9.26	2.12		15.62	4.96		8.90	2.59	
≥30 Obesity	24	7.92	3.31		13.21	5.07		8.79	2.50	
Regular gynecological follow-up				0.04			0.05			0.02
No	50	8.82	2.64		15.28	4.90		8.28	2.77	
Yes	463	9.48	2.07		16.67	4.67		9.12	2.40	
Breast self-examination				0.02			0.01			<0.01
No	277	9.21	2.27		16.00	4.69		8.75	2.53	
Yes	233	9.65	2.02		17.17	4.66		9.37	2.30	
Depressive symptoms (score CES-D ≥ 23) (Q_D15_)				0.02			<0.01			<0.01
No	405	9.29	2.14		16.93	4.43		9.26	2.33	
Yes	88	9.91	2.31		14.82	5.64		8.26	2.76	
*BRCA1/2* mutation				0.13			0.36			0.08
Non carriers	293	9.27	2.20		16.34	4.61		8.86	2.49	
Carriers	224	9.56	2.12		16.72	4.82		9.24	2.39	

CES-D: Center for Epidemiologic Depression Scale; Q_D15_: questionnaire filled in 15 days after test result disclosure.

## Discussion

The aim of the present study was to investigate the psychometric properties of a generic body image scale originally developed by LN Lodder [22] on a population of unaffected female *BRCA1/2* mutation carriers / non carriers. As far as we know, this is the first study in which the psychometric properties of this scale have been studied, although it has been used by the authors of epidemiological studies. First, the Body and Breast Image Scale (BBIS) turned out to have good psychometric properties for assessing both generic body image and specific breast image in unaffected French women. Secondly, the results showed that the BBIS is a three-dimensional instrument. All the items in the scale should not be combined into an overall score: three scores should be calculated, based on the three dimensions “ValBI”, “SatBIPA” and “SatBr” which were brought to light here. Thirdly, analysis of the associations involving the BBIS scores showed the existence of (i) no associations with the respondents’ socio-demographic data (age, level of education, living with a partner), (ii) no associations with *BRCA1/2* mutation carrier status, (iii) associations with the respondents’ clinical characteristics (body mass index, regular gynecological follow-up, breast self-examination, depressive symptoms).

First, the BBIS showed satisfactory psychometric properties. The incomplete response rate to the BBIS was only about 9% (1.6% to 3.9% depending on the items). In the multivariate analysis, the only variable found to be significantly associated with a lower risk of missing values was a high level of education, which is known to be a predictive factor of fewer missing values in epidemiological studies [33]. A ceiling effect was observed with the eighth and ninth items, possibly due to their negative wording [34]. The ninth item (“I had difficulty touching my breasts”) showed the strongest ceiling effect, but we decided to keep it because it might be sensitive to change among carriers of a *BRCA1/2* mutation, who might eventually opt for prophylactic surgery. However, this ceiling effect was not observed any longer when the various items in each dimension in which it was observed were pooled.

The consistency of the sub-scales was moderate to good. The moderately low value of the coefficient obtained on the “SatBr” dimension may have been due to the small number of items in this sub-scale. However, a Cronbach’s alpha coefficient of 0.50 or more can be taken to suffice in an exploratory analysis of this kind [34]. Some items (i.e. items 12 & 8) had similar loadings on several sub-scales. However, based on the meaning of these items and on the fact that the item convergent validity and item discriminant validity criteria were satisfied, these two items were taken to belong to the “SatBIPA” dimension.

Secondly, based on the results of an exploratory factorial analysis, the BBIS was found to be a three-dimensional scale. The factor analysis and the goodness-of-fit indices confirmed this finding. Upon analyzing the three scores based on the three dimensions, it was observed that they could vary differently, depending on some respondents’ characteristics, emphasizing that it was necessary to analyze each dimension independently, as they all reflected different concepts. As far as we know, LN Lodder was the first author to analyze responses to the BBIS by drawing up two scores, one on the general body image and one on the breast-related body image [22], but no statistical analysis of the dimensional structure of the scale was presented in that study. Judging from our results, Lodder’s general body image dimension actually consisted of two dimensions (“ValBI” reflecting a body image trait and “SatBIPA” reflecting a body image state) [35].

Thirdly, the results obtained upon examining the relationships with various characteristics were consistent with data previously published in the literature. No significant associations were found to exist between the scores obtained on the BBIS and the socio-demographic variables collected, including age. Since marked changes in appearance occur during adult life, especially in women, one might expect the body image to undergo similar changes. In fact, body dissatisfaction has been found to remain unchanged during the whole life span in women as the importance of women’s body shape, weight, and appearance decreases. An important distinction therefore has to be made between self-assessments and the importance of the body in general [36]. The level of education was not found here to be a determinant. Some studies have shown that individuals with a higher socioeconomic status, especially women, tend to be more dissatisfied with their bodies than those with a lower status [37-39]. It has been suggested that this might be due to the role played by thinness as an indicator of social status [38]. Although the scores obtained here were not significantly associated with the level of education, they showed the existence of a tendency on these lines. Living with a partner was not found to be a significant determinant. It has been reported that marital status is not associated with body dissatisfaction, although low marital satisfaction was found to be significantly associated with body dissatisfaction [40-42].

We did not find any significant links between our measures and the respondents’ *BRCA1/2* mutation carrier status. However, there was a trend indicating that differences may exist between *BRCA1/2* mutation carriers and non-carriers as regards breast satisfaction. One might expect breast satisfaction to be lower among *BRCA1/2* mutation carriers, who may feel uncomfortable about their breasts, which they may regard as a source of disease. Our results unexpectedly showed the existence of a trend whereby higher scores were obtained on the “SatBr” dimension among *BRCA1/2* mutation carriers. Disclosure of positive test results was therefore not associated with a poorer breast image. It would now be worth assessing the effects of mutation carrier status and those of prophylactic surgery on *BRCA1/2* mutation carriers with time [22,23].

The present results showed the existence of significant associations between our body image scores and the respondents’ clinical characteristics. The links between high BMI and poor body satisfaction have been widely documented [13,42,43]. The lack of associations observed here between the BMI and the “SatBr” scores was more unexpected. This finding illustrates the fact that differences can exist between body image and breast image, depending on the BMI class, and thus shows that the breast-related body image should be assessed independently.

Significant associations were found to exist between the BBIS scores obtained here and the respondents’ depressive symptoms; higher scores were obtained on the dimension “ValBI” but lower scores on the dimensions “SatBIPA” and “SatBr” by women with depressive symptoms. Body image is partly based on beauty norms and the ideal body, and people assess their body partly depending on how well it matches their picture of the ideal body. Depressive symptoms are associated with low self-esteem, which can result from the existence of a large discrepancy between one’s perceived body and one’s ideal body. The present results confirm the existence of a gap between the ideal, imaginary body (as assessed by the importance attached to body image, “ValBI”) and the perceived body (“SatBIPA” and “SatBr”) in people with psychological disorders [44]. The links between body image dissatisfaction and depressive symptoms or psychological disorders have been previously documented in many clinical and non clinical contexts [37,42,45,46].

One of the limitations of this study on the psychometric properties of the scale tested was that the relationships between this scale and other similar concepts were not explored*.* Since the data collected in the framework of the GENEPSO cohort included only one body image scale, it was not possible to assess concurrent validity criteria. However, our results on the associations between our BBIS measurements and various respondents’ characteristics were consistent with data available in the literature. Another limitation of this study was the relatively low reliability of the “SatBr” subscale. However, the low value obtained in this respect, which may have been partly due to the small number of items in the subscale, was nevertheless higher than a threshold proposed for exploratory analyses [34]. The last limitation might be the non-random selection of our study population. But as the women included in this multicenter study were recruited just before the consultation at which they received their test results, our sample can be said to be a non-selected sample representative of the healthy women tested for *BRCA1/2* mutations at French cancer genetic clinics.

One question which needs to be discussed is whether the results obtained here can be generalized and whether the scale presented here can be used in other contexts. This scale can obviously be used only on women, as it focuses on several aspects relating to femininity and breasts. The characteristics of our study population were compared with those of women in the French general population, based on data published by INSEE [47] (the National Institute for Statistics and Economic Science): the women in our study differed from those in the general population in that they were younger and had a higher level of education. But they resembled the women in the general population in terms of BMI and the presence of depressive symptoms. As the women’s socio-demographic characteristics, contrary to their medical characteristics, were not associated with the results obtained on the BBIS, our results can presumably be generalized to French women on the whole. As far as the use of the scale in other countries is concerned, special attention should be paid to societal ideals and cultural norms, as these factors can greatly influence body perception.

## Conclusions

One of the main strengths of the Breast and Body Image Scale (BBIS), a three-dimensional scale allowing to assess three components of body image in women, is that it was not drawn up with any particular pathology or predisposition to any specific disease in mind. It can therefore presumably be applied to various female populations, whether they are healthy or suffer from a specific disease. The BBIS can therefore be said to be a means of obtaining a basic generic picture of body image, to which users could add further items (organ-related items, disease-related items, etc.) when assessing body image in specific contexts. The BBIS has several potential applications. It could be used, for example, to assess the changes with time in respondents’ body image in various contexts such as longitudinal epidemiological studies or clinical trials. In the context of *BRCA1/2* carriers, it could be used to assess the impact of prophylactic surgery (*i.e.* risk-reducing mastectomy and/or oophorectomy) on body image with time.

This study is the first step in the validation of the BBIS, a generic body image scale. Further research is now required to confirm the reliability of the findings obtained here by applying this tool to other populations in other contexts.

## Competing interests

The authors declared that they have no competing interest.

## Authors’ contributions

NR performed all statistical analyses and wrote the first draft of the manuscript. CN conceived the GENEPSO cohort and supervised data collection. RG contributed to methods and interpretation of data. CJR designed the study, contributed to methods, analysis and interpretation to the data, and participated in drafting the manuscript. All the authors have read, commented and approved the final version of the manuscript.

## Pre-publication history

The pre-publication history for this paper can be accessed here:

http://www.biomedcentral.com/1472-6874/13/24/prepub
